# Using Slow-Paced Breathing to Foster Endurance, Well-Being, and Sleep Quality in Athletes During the COVID-19 Pandemic

**DOI:** 10.3389/fpsyg.2021.624655

**Published:** 2021-05-13

**Authors:** Uirassu Borges, Babett Lobinger, Florian Javelle, Matthew Watson, Emma Mosley, Sylvain Laborde

**Affiliations:** ^1^Department of Performance Psychology, Institute of Psychology, German Sport University Cologne, Cologne, Germany; ^2^Department of Social and Health Psychology, Institute of Psychology, German Sport University Cologne, Cologne, Germany; ^3^Department for Molecular and Cellular Sports Medicine, Institute for Cardiovascular Research and Sports Medicine, German Sport University Cologne, Cologne, Germany; ^4^Department of Sport Science and Performance, Solent University, Southampton, United Kingdom; ^5^UFR STAPS, Université de Caen Normandie, Caen, France

**Keywords:** HRV, cardiac vagal activity, cholinergic anti-inflammatory pathway, cytokine storm, mental health, lung inflammation, biofeedback

## Abstract

The coronavirus disease 2019 (COVID-19) has been causing major disruptions in the sporting world. Negative physiological and psychological effects on athletes have been reported, such as respiratory issues and increased stress. Therefore, it is timely to support this population by presenting cost-effective and accessible intervention techniques to reduce this impact. Slow-paced breathing (SPB) has the potential to counteract many of the detrimental effects of COVID-19 that can directly affect sports performance. In this article, we present and justify the use of SPB in athletes by focusing on three key outcomes, namely aerobic endurance performance, emotional well-being, and sleep quality. We examine the physiological mechanisms that underpin these three outcomes and review literature showing that SPB can activate anti-inflammatory pathways, increase lung capacity and, in turn, improve aerobic endurance, emotional well-being, and sleep quality. We conclude that interventions using SPB can have preventive and rehabilitative properties for athletes. Future studies should empirically test the potential of SPB to help this specific population.

## Introduction

The global outbreak of the coronavirus disease 2019 (COVID-19) in March 2020 (WHO, 2020) is having a major negative impact on the physiological and psychological health of individuals and society in general (Brooks et al., 2020; Fu et al., 2020; Mohanty et al., 2020). Physiological effects range from dyspnea (Carfì et al., 2020) to acute myocardial injury (Zheng et al., 2020) whilst negative psychological effects include impairments in well-being and mental health (Hull et al., 2020). These negative effects are of concern in professional sport because they are likely to be detrimental to athletic performance, thus it is important to ensure that athletes are receiving the appropriate support during the outbreak (Hull et al., 2020). Accordingly, the aim of this article is to propose the use of slow-paced breathing (SPB) in athletes to counteract the negative physiological and psychological impacts of COVID-19.

The majority of symptomatic patients have mild flu-like symptoms. However, the physiological effects associated with severe acute respiratory syndrome coronavirus 2 (SARS-CoV-2) infections can evolve into pulmonary embolism, pulmonary fibrosis, acute myocardial injury, and chronic damage to the cardiovascular system (Faggiano et al., 2020; Zheng et al., 2020; McDonald, 2021; Raman et al., 2021). Little is known about the long-term sequelae of COVID-19 as a result of these complications. A study of 147 symptomatic COVID-19 patients showed that only 18 (12.6%) were completely free of any COVID-19-related symptoms around 2 months following symptom onset (Carfì et al., 2020). In another study, almost half of recovered patients reported at least one unresolved symptom related to COVID-19 (Klein et al., 2021). Aside from the impairment of certain physiological functions, other negative economic, social, and psychological consequences can be observed, such as infection fears, financial loss, frustration, decreased mood, and post-traumatic stress symptoms (Brooks et al., 2020; Huang and Zhao, 2020). In general, mental health issues have been common during the COVID-19 outbreak (Huang and Zhao, 2020; Shi et al., 2020). In line with this, 44.1% of patients recovering from COVID-19 reported worsened quality of life around 2 months following symptom onset (Carfì et al., 2020).

Although there is no empirical evidence on the prevalence or impact of COVID-19 on physiological processes in athletes to date, the potential for COVID-19 to impair performance is highly concerning for professional athletes (Kim et al., 2020). Literature on COVID-19 has raised concerns that athletes may be more susceptible to viral respiratory tract infection due to vigorous exercise (Hull et al., 2020). Regarding psychological issues, young people—a demographic that applies to most athletes—represent a high-risk group for mental illness in the COVID-19 pandemic (Huang and Zhao, 2020; Shi et al., 2020). The potential mid- to long-term sequelae due to the infection caused by the SARS-CoV-2, the postponement of major sports events such as the Olympic Games in Tokyo, and social distancing may constitute major health- and career-related stressors for athletes (Hull et al., 2020). These stressors negatively affect outcomes at different levels, for instance endurance, mood, and sleep quality (Mon-López et al., 2020). Decreased aerobic endurance and emotional well-being can be decisive in competitive situations, especially when athletes strive to achieve peak performance under pressure. However, there is a lack of interventions specific to the context of sports within a pandemic period that (1) target both physiological and psychological issues and (2) can easily be implemented into daily life. It is timely to develop strategies to counteract the negative impact of COVID-19 in sports and support athletes’ performance and mental health.

In this perspective article, we propose that SPB is an intervention technique that can have preventive and rehabilitative effects on certain sports-relevant outcomes that are impaired by COVID-19, namely aerobic endurance, emotional well-being, and sleep quality. In the following subsections, we (1) review literature on the effect of SPB on the psychophysiological processes that are negatively affected by COVID-19, and which are especially relevant for the aforementioned outcomes, and (2) outline the working mechanisms of SPB that may underpin these effects. Regarding preventive effects, we argue that SPB may contribute to less mid- to long-term physiological sequelae in athletes when they are eventually infected with SARS-CoV-2. This reduction of sequelae is thought to occur because of the health-promoting effects of SPB, which can strengthen psychophysiological processes involved in aerobic endurance performance, emotional well-being, and sleep quality. Regarding rehabilitative effects, we argue that SPB may reduce the detrimental effects of COVID-19 on sports performance, thus potentially shortening the length of the recovery period.

## Slow-Paced Breathing

Slow-paced breathing is a breathing technique that aims to slow down the inhalation and exhalation phases to a controlled pace that is determined by a visual, auditory, or kinesthetic pacer (Laborde et al., 2019a). Whereas spontaneous breathing usually comprises between 12 and 20 cycles per minute (cpm) in adults (Tortora and Derrickson, 2014), SPB reduces the breathing pace to around 6 cpm (Lehrer and Gevirtz, 2014). The mechanisms underlying the positive physiological effects of SPB are the following: Firstly, it enhances respiratory sinus arrhythmia, i. e., the increase and decrease in heart rate concomitant to, respectively, inhalation and exhalation (Lehrer and Gevirtz, 2014; Shaffer and Meehan, 2020). Secondly, it enhances baroreflex functioning, combining respiratory frequency to the intrinsic frequency of the baroreflex, which is also 6 cpm (Lehrer and Gevirtz, 2014). Finally, it enhances vagal afferents within the inhalation phase via stretching of the pulmonary afferents (Noble and Hochman, 2019) and enhances vagal efferents in both the short and long term (Laborde et al., 2019a). Importantly, the vagus nerve seems to underlie the range of physiological effects influenced by SPB (Bernardi et al., 2001; Gerritsen and Band, 2018; Mather and Thayer, 2018; Shaffer and Meehan, 2020).

Slow-paced breathing has already been shown to have positive effects on both physiological and psychological levels (Lehrer et al., 2020). In athletes, interventions that slow breathing rate (i.e., through biofeedback or SPB) can improve emotional well-being (Mather and Thayer, 2018; Laborde et al., 2019a; Lehrer et al., 2020), improve sleep quality (Laborde et al., 2019a), and positively influence athletic performance both objectively and subjectively (Jiménez Morgan and Molina Mora, 2017). In the following subsections, we review current literature the sports-relevant impairments caused by SARS-CoV-2 that may be counteracted by SPB and summarise these findings in Table 1.

**TABLE 1:** Summary of studies with slow-paced breathing that target variables that are relevant in the context of the COVID-19 pandemic.

Study	Relevant dependent variable	Relevant findings with effect sizes^1^	Biofeedback	Target population	Sample size
Bernardi et al., 2001	Hypoxia, hypercapnia, and baroreflex sensitivity	SPB reduced the chemoreflex response to both hypoxia and hypercapnia	No	Healthy adults	15
Bernardi et al., 1998	Oxygen saturation and exercise performance	SPB reduced dyspnea and improved both resting pulmonary gas exchange and exercise performance	No	Chronic heart failure patients and healthy adults	61
Bilo et al., 2012	Ventilation efficiency for oxygen	SPB increased blood oxygenation and reduced systemic and pulmonary arterial pressure	No	Healthy adults	Experiment 1: 39; Experiment 2: 28
Laborde et al., 2019a	SSQ and CVA	SPB increased subjective sleep quality (*d* = 0.51) and increased overnight-CVA (*d* = 0.68) as well as morning-CVA (*d* = 0.42)	No	Healthy adults	64
Laborde et al., 2019b	Adaptation to psychological stress after physical exertion and CVA	SPB led to better Stroop interference accuracy (ηp^2^ = 0.170) after physical exertion	No	Young adults	Experiment 1: 60; Experiment 2: 60
Laborde et al., 2016	CVA	CVA was higher during SPB compared to control (*d* = 0.35)	No	Adolescents with intellectual disability	17
Paul and Garg, 2012	Anxiety, CVA, and sports performance	SPB reduced both trait and state anxiety, increased CVA (HF), and increased sports performance (dribbling, shooting, and passing)	Yes	Basketball players	30
Pusenjak et al., 2015	Self regulation (heart rate, galvanic skin response, and coherence)	HR during stress tasks was better in SPB group than control.	Yes	Athletes	39
Wells et al., 2012	CVA and stress reaction	SPB after stressor increased HF (ηp^2^ = 0.122)	Yes	Trained musicians	45

*Notes. ^1^Wherever reported. CVA, cardiac vagal activity; SPB, Slow-paced breathing; SSQ, Subjective sleep quality; HRV, Heart rate variability; and HF, High frequency.*

### Affected Processes Linked to Cardiac Vagal Activity Modulation

Symptomatic COVID-19 patients can develop Acute Respiratory Distress Syndrome (ARDS). ARDS is characterized by a strong immune response that involves the release of high levels of various pro-inflammatory cytokines in the lungs, a phenomenon known as cytokine storm or hyperinflammation (Bonaz et al., 2020; Guo et al., 2020). Hyperinflammation may be caused by the cytopathic effect mediated by SARS-CoV-2 expressing the binding spike-like surface projections for the angiotensin-converting enzyme 2 (ACE2) receptor. This virus enters the target organ cell by binding its spikes to ACE2, a membrane-bound aminopeptidase that is highly expressed in the heart and lungs. The inhibition of the ACE2 function contributes to hyperinflammation (Fudim et al., 2020). This hyperinflammation can lead to apoptosis of epithelial cells as well as to vascular leakage, which results in ARDS (Martin et al., 2005). The interaction of SARS-CoV-2 with ACE2 may also lead to chronic damage to the cardiovascular system (Kaniusas et al., 2020; Zheng et al., 2020).

It has been hypothesized that hyperinflammation and the consequent worsening of a patient’s health status can be reduced or even prevented by specifically targeting the cholinergic anti-inflammatory pathway (Bonaz et al., 2020). Pro-inflammatory cytokines activate the afferent fibers of the vagus nerve, the major nerve of the parasympathetic nervous system. The afferent vagal fibers transport inflammatory information to the nucleus of the solitary tract, which enables the detection of the inflammatory process. The neurons of the nucleus of the solitary tract activate the dorsal motor nucleus of the vagus nerve, whose efferent fibers trigger the cholinergic anti-inflammatory pathway (Pavlov et al., 2003; Kaniusas et al., 2020). The cholinergic anti-inflammatory response is characterized by the activity of acetylcholine, which is secreted by T-cells when they identify macrophages or other cytokine-producing cells (Kaniusas et al., 2020). Acetylcholine binds to the acetylcholine surface receptors of macrophages, which inhibits the release of inflammatory mediators by these macrophages and leads to the suppression of pro-inflammatory cytokines (Kaniusas et al., 2020).

To summarize, the vagus nerve modulates the immune response to inflammatory processes in the body by means of the cholinergic anti-inflammatory pathway, which counteracts the cytokine storm caused by ARDS (Kaniusas et al., 2020). In support of this, a meta-analysis showed that heart rate variability (HRV) indices of cardiac vagal activity (CVA)—the activity of the vagus nerve regulating cardiac functioning—are negatively associated with markers of inflammation (Williams et al., 2019). This finding suggests that CVA serves as an indirect marker of the activity of the neurophysiological function responsible for adaptively regulating inflammatory processes in humans (Williams et al., 2019). Consequently, increasing CVA can lead to increased anti-inflammatory effects, which reduces the severity of COVID-19.

Slow-paced breathing has already been shown to enhance CVA (Wells et al., 2012; Laborde et al., 2019a; Lehrer et al., 2020), with evidence existing for acute (Laborde et al., 2016; You et al., 2021), and chronic (Laborde et al., 2019a) increases in CVA. In athletes, SPB acutely increased CVA even after one single SPB session (You et al., 2021). CVA has been used as a biofeedback marker for SPB training in the applied field (Lehrer and Gevirtz, 2014; Shaffer and Meehan, 2020). A recent meta-analysis showed that SPB combined with HRV biofeedback has the largest effect sizes for athletic performance in general (Jiménez Morgan and Molina Mora, 2017; Lehrer et al., 2020; Pagaduan et al., 2020), and another with non-athletes revealed that SPB with HRV biofeedback reduced self-reported acute stress and anxiety with large effect size (Goessl et al., 2017). Three theories, namely the neurovisceral integration model (Thayer et al., 2009), the polyvagal theory (Porges, 2007), and the vagal tank theory (Laborde et al., 2018b), highlight the role of CVA as a marker of the effectiveness of self-regulatory processes and health-related autonomic regulation. SPB, with or without biofeedback, has shown to increase adaptive self-regulation, for instance inhibition (Laborde et al., 2019a), and to counteract self-regulation failure, by lowering stress (Laborde et al., 2016) and anxiety (Paul and Garg, 2012; Wells et al., 2012), including competitive anxiety (Pusenjak et al., 2015). Consequently, SPB is thought to strengthen the connection between brain networks involved in emotion regulation (Mather and Thayer, 2018), thus contributing to increased emotional well-being. In general, the main mechanisms of action underlying the physiological and psychological effects of SPB are expected to derive from this influence of SPB on CVA, as already discussed (Lehrer and Gevirtz, 2014; Jiménez Morgan and Molina Mora, 2017). As increased CVA may prevent cytokine release syndrome (Huston and Tracey, 2011) and has been positively associated with both athletic performance (Buchheit, 2014; Bellenger et al., 2016; Jiménez Morgan and Molina Mora, 2017; Lehrer et al., 2020; Pagaduan et al., 2020) and emotional well-being (Mather and Thayer, 2018), we argue that CVA mediates the benefits of SPB on the sports-relevant outcomes that can be impaired by COVID-19 which we describe in this article.

### Affected Processes via Lung Function Modulation

Respiratory issues are among the most common concerns related to COVID-19 and they can occur in the form of diffuse alveolar damage, severe hypoxemia, interstitial pneumonia, or increased pulmonary embolism (Faggiano et al., 2020; Kaniusas et al., 2020; Mohanty et al., 2020). Dyspnea has been found to be one of the most prevalent manifestations to persist in patients following recovery from symptomatic COVID-19, with most patients reporting symptoms for as long as two (Carfì et al., 2020; Rodriguez-Morales et al., 2020; Raman et al., 2021) to 3 months (Arnold et al., 2020; Garrigues et al., 2020; Wong et al., 2020) after discharge from hospital. As previously described, SARS-CoV-2 targets ACE2 receptors, which are highly expressed in the lungs. The virus then invades alveolar epithelial cells, resulting in the aforementioned respiratory symptoms (Turner et al., 2004; Zheng et al., 2020). Indeed, pulmonary fibrosis, a scarring of the lung tissue that most commonly presents as dyspnea, has been found three months after onset of COVID-19 (Zha et al., 2020; Willi et al., 2021). Given that restrictive ventilator defects caused by the pulmonary fibrosis can substantially affect patients’ physical abilities in the recovery stage, early rehabilitation seems particularly important (Yu et al., 2020).

We propose that SPB is an effective technique to improve lung function for the purpose of both prevention and rehabilitation. The effects of SPB on lung function are explained by means of the biomechanics of lung ventilation, which is strictly related to blood oxygen, carbon dioxide, and pH homeostasis (Russo et al., 2017). SPB leads to lower respiratory rate and increased tidal volume (Bernardi et al., 2001; Russo et al., 2017). Decreasing respiratory rate and increasing tidal volume improves ventilation efficiency by reducing alveolar dead space, thus improving oxygen saturation (Bilo et al., 2012). SPB has been shown to represent the optimal breathing rate for improving alveolar ventilation and reducing dead space, which in turn increased general exercise performance in a study of chronic heart failure patients (Bernardi et al., 1998). Because of the potential of SPB to improve oxygen saturation in different populations, we argue that SPB may help limit the impact of the shortness of breath caused by pulmonary fibrosis, thus enhancing aerobic endurance performance during exercise.

### Effects on Sleep Quality

An array of recent studies have shown that the COVID-19 pandemic has a detrimental impact on sleep quality, with low sleep quality leading to higher daily dysfunctions (Cellini et al., 2020; Fu et al., 2020; Salehinejad et al., 2020; Targa et al., 2020). In general, the changes in social and work-related schedules as a result of COVID-19-related restrictions lead to alterations in sleep patterns and individuals’ circadian rhythms (Targa et al., 2020), however, the effects of these changes in athletes specifically is still unknown. The cancelation or postponement of competitions, for instance the Olympic Games in Tokyo (Olympic Games, 2020), may also impair professional athletes’ sleep quality due to the inherent uncertainty and disruption of medium- to long-term goals. As sleep restriction can negatively affect athletic performance, cognitive function, and mood (Fullagar et al., 2015), research into the impact of the COVID-19-pandemic restrictions on athletes’ sleep quality is warranted.

Due to its restorative effects on the immune and endocrine systems (Frank and Benington, 2006), sleep is critical for professional athletes during the COVID-19 pandemic (Bonaz et al., 2020). Sleep disturbance impairs immunity via activation of the hypothalamic-pituitary-adrenal axis and the sympathetic nervous system (Peake et al., 2017). A nocturnal sleep pattern has been shown to prime the immune system to challenge infections challenge and enhance immune-surveillance. When sleep is disrupted, the activity of T helper 1 cytokines such as interferon-γ, which provide important protection against intracellular viral and bacterial change, tends to decrease, whereas the activity of T helper 2 cytokines such as interleukin-10 tends to increase (Irwin, 2015). Over time, the decreased activity of T helper 1 cytokines can lead to a detrimental host defense, which is also characterized by an increase in the plasma inflammation markers C-reactive protein and interleukin 6 (Peake et al., 2017). Studies have demonstrated the detrimental effects of low sleep quality on immunity by, for example, showing a decreased anti-hepatitis antibody response after hepatitis-B vaccination (Prather et al., 2012), and an increased chance of developing a common cold (Prather et al., 2015).

To date, few published studies have investigated the effect of SPB on sleep quality (Tsai et al., 2015; Laborde et al., 2019a). One, an intervention study with self-reported insomniacs, showed improved markers of sleep quality such as sleep efficiency when participants practiced SPB for 20 min before going to sleep, although vagally-mediated HRV parameters did not differ from spontaneous breathing (Tsai et al., 2015). Another study showed that, following a 30-day SPB intervention, subjective sleep quality was higher in the SPB group when compared to a control group, with this result being mediated by increased CVA (Laborde et al., 2019a). Although more research on the effects of SPB on sleep quality is needed, these initial findings point toward the potential of SPB for improving sleep quality. We argue that increased sleep quality, along with increased CVA, will improve immunity in athletes, thus contributing to increase aerobic endurance and emotional well-being.

### Considerations for Slow-Paced Breathing Interventions

Given the potential benefits of SPB on the aforementioned mechanisms and outcomes, appropriately conducted intervention studies are of critical importance in order to test thepsychophysiological effectsof SPB on athletes. Ideally, an implementation of an SPB interventionduring a COVID-19-pandemic adheres to at least three key principles:1). It is delivered in a “COVID-secure” manner, (2) it is easy to incorporate into the individual’s daily routine, and (3) it is cost-effective. Oneway to realize all three of these principlesis through a smartphone-guided breathing application. Athletes could be educated through a virtual workshop to learn the correct breathing technique prior to downloading a related smartphone application. Smartphone applications that display and time the duration of the in hale and exhale within an SPB exercise have been shown to be effective in increasing CVA (Laborde et al., 2019a), whilst applications more generally are also useful for increasing the accessibility of mental skills training for athletes (Rist and Pearce, 2017). Certainly, smartphone applications can overcome many of the barriers that may have prevented in-person interventions from taking place, such as the current social and travel restrictionsas well associal distancing rules. Of the many methods to enhance CVA (Fatisson and Oswald, 2016; Laborde et al., 2018a), for instance transcutaneous vagus nerve stimulation (Borges et al., 2019, 2020, 2021), or meditation with mindfulness training (Garland et al., 2014), SPB offers a simple, easy-to-implement, and beneficial intervention technique with both psychological and physiological outcomes for athletes that can be delivered in lockdown or isolation scenarios.

Previous work has found that aneffective way to integrate SPB into one’s daily routine is to perform the exercise before going to sleep (Laborde et al., 2019a). This daily ‘event’ can representa simple prompt by which to remember to complete the SPB session, whilst itis also usually a quiet time of the day. It is particularly suitablewhen considering that SPB may acutely increase sleep quality (Laborde et al., 2019a). Interestingly, there is considerable variation in the implementation of SPB in research. For instance, it has been performed with or without biofeedback or a resonance assessment, and convenient protocols can be found in literature (Lehrer et al., 2000; Shaffer and Meehan, 2020). We recommend a daily SPB session of 15minutes,breathing at a rate of 6 cpm witha slightly longer exhalation (through pursed lips) than inhalation (through the nose). Prolonged exhalation has been shown to contribute to larger fluctuations in beat-to-beat heart rate compared to prolonged inhalation and, therefore, to higher CVA (Strauss-Blasche et al., 2000; Allen and Friedman, 2012; Laborde et al., 2016, 2019a). Slower breathing can be difficult for some individuals, which may lead to hyperventilation and expelling excessive amounts of carbon dioxide (Shaffer and Meehan, 2020). In such cases, the participants should be instructed to take shallower and smoother breaths whenever they feel faint or that their heart is beating too hard (Shaffer and Meehan, 2020). Alternatively, SPB may be contraindicated for athletes whose overbreathing compensates for increased acidity in the blood. For these individuals, SPB may increase CO_2_ levels in the blood and dangerously increase acidosis (Khazan, 2013; Shaffer and Meehan, 2020).

Future studies on the effects of SPB on the variables discussed here should perform SPB either within a single testing session (Laborde et al., 2019b; You et al., 2021) or following a number of daily sessions, for instance over four to 8 weeks (Schumann et al., 2019; Laborde et al., 2019a). In both cases, the variables of interest should be measured before and directly after the intervention. If the aim of the respective study is to investigate mid-term effects, then measurements should also be made after a number of weeks. A longside the SPB condition, the prospective study should have at least one control condition that involves participants performing a comparable task, which can be using social media or performing SPB with a frequency within the range of spontaneous breathing, i.e., between 12 and 20 cpm. Previous research investigating SPB shown effect sizes of *d* = 0.51 (subjective sleep quality) and partial η^2^ = 0.20 (for CVA) when compared with a control condition (Laborde et al., 2019a), equivalent to medium and large effects, respectively, with a sample size of 64 participants (Cohen, 1988).

## Discussion

**FIGURE 1:**
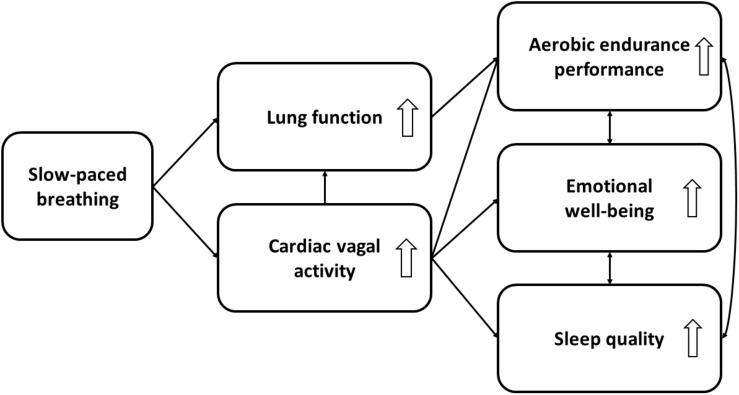
Expected mechanisms of action between slow-paced breathing and aerobic endurance performance, emotional well-being, and sleep quality. White arrows depict the direction of the effect [i.e., either an increase (up) or decrease (down) of the psychophysiological processes].

In this perspective article, we have discussed the potential of using SPB to strengthen psychophysiological processes that can be impaired by COVID-19, thus counteracting its negative impact on sports performance. We have focused on three variables that are particularly important for sports performance, namely aerobic endurance performance, emotional well-being, and sleep quality. The processes and possible mechanisms of action discussed here are depicted in Figure 1.

To summarize, we expect the following mechanisms of action to underpin the positive effects of SPB on aerobic endurance performance: Firstly, SPB enhances CVA, which is an index of anti-inflammatory effects regulated by the activity of the vagus nerve (Williams et al., 2019). Secondly, in parallel to the beneficial effects of CVA on lung function, SPB improves oxygen saturation, thus enhancing lung function and limiting the effects of dyspnea caused by COVID-19 (Bilo et al., 2012; Russo et al., 2017). Thirdly, sleep quality, which is increased as a result of the positive effect of SPB on CVA (Laborde et al., 2019a), improves immunity (Frank and Benington, 2006), thus helping the body to resist the COVID-19 infection (Targa et al., 2020). A stronger general anti-inflammatory function, coupled with greater oxygen saturation and immunity, may contribute to increased aerobic endurance performance. Future empirical research that seeks to test these mechanisms is strongly recommended as no such studies currently exist. Regarding emotional well-being, SPB can increase CVA and potentially emotional well-being. CVA is positively associated with emotion regulation (Thayer et al., 2009; Mather and Thayer, 2018), which may explain the role of SPB in reducing stress and anxiety, thus increasing emotional well-being (Goessl et al., 2017). Additionally, SPB promotes emotional well-being through improving sleep quality, as sleep positively affects mood (Fullagar et al., 2015).

Understanding the aforementioned mechanisms of action underlying SPB enables the generation of preventive and rehabilitative intervention ideas for professional athletes. The target group for SPB as a preventive measure consists of athletes without any COVID-19 infection history. SPB for this group is expected to serve a protective function, as the COVID-19-relevant physiological processes may be optimized by such an intervention. Thus, athletes without any COVID-19 history will be less susceptible to a COVID-19 infection, or will have a reduced likelihood of presenting complications in the event of a future infection. Future research should investigate the effects of SPB on COVID-19 or further diseases that cause respiratory syndromes. Regarding rehabilitative effects, SPB is expected to be effective in athletes who have already been infected. As the physiological processes that are relevant for sports performance are thought to be optimized, SPB may have the potential to speed up the recovery process, thus enabling the athletes to return to training more rapidly. Future studies should investigate this hypothesis in athletes with a medical history of COVID-19. In general, the effectiveness of both preventive and rehabilitative approaches should be examined.

Aside of testing both preventive and rehabilitative approaches, the mechanisms described in this article enable future research to test further new hypotheses. Firstly, intervention studies of SPB in athletes should be conducted to examine the benefits of this technique as proposed here. Secondly, considering the societal impact of COVID-19, the model presented in Figure 1 is able to accommodate additional variables derived from future research or be transferred to other fields with different target groups. The general population, which usually has less optimal CVA (Rossi et al., 2014) and lung function (Stang et al., 2015) in comparison to athletes, may particularly benefit from SPB interventions and show greater improvements in these physiological outcomes.

## Conclusion

Slow-paced breathing can activate anti-inflammatory pathways and increase lung capacity, which consequently increases aerobic endurance, emotional well-being, and sleep quality. Its cost-effectiveness and ease of use makes this technique a promising way to counteract the symptoms of COVID-19. Thus, SPB may become an essential part of the toolbox of sports psychologists in times of widespread restrictions, uncertainty, and possible performance decrements.

## Data Availability Statement

The original contributions presented in the study are included in the article/supplementary material, further inquiries can be directed to the corresponding author/s.

## Author Contributions

UB and SL contributed to the conceptualization of the original draft. UB wrote the first draft. SL, BL, FJ, EM, and MW provided critical comments to improve the manuscript. All authors contributed to the article and approved the submitted version.

## Conflict of Interest

The authors declare that the research was conducted in the absence of any commercial or financial relationships that could be construed as a potential conflict of interest.
